# 

**Published:** 1819-01-01

**Authors:** 


### CORRESPONDENCE.

The valuable Communication from Dr. David Davies, of Bristol, will certainly appear in our next number.

The interesting papers from Mr. Johnson, of Torrington, in Devonshire, are received, and shall have early attention, in the way he alludes to. Mr. Bushel's curious chemical phænomenon is received. Dr. Carbutt's very valuable Medical Cases are unavoidably postponed till next number; as is the highly important document from Dr. Dewar, relative to the wounded in the battle of Algiers. Mr. Colville's Case of Spontaneous Cure of Ascites, and Mr. John Hall's Case of fractured Spine from jumping into the Sea, are unavoidably postponed.

Mr. Grabham's Case of Monstrosity in our next; as will, if possible, Dr. Porter's Cases of Erysipelas Phlegmonalis.

In consequence of the important paper of "Atticus," Dr. M'Whirter's Communication, transmitted through the Army Medical Board, was obliged to be postponed till next number.

An answer to Juvenis Studens in our next.

Mr. Sprague's promised favours will always be acceptable.

The readers of Mr. Guthrie's paper in the Original Department of the Journal are particularly *referred* to Vol. viii of the Medico-Chirurgical Transactions; or Vol. v, pages 510—11—12 of this Journal, for several judicious observations and restrictions relative to the employment or non-employment of mercury in syphilitic complaints.

---

### TO THE MEDICAL PROFESSION.

The ardour which pervades the world of Medical Science at this moment; the number of new and important publications which are daily issuing from the press; and the rapidly increasing list of correspondents, all render it imperative on us to enlarge the limits of our Journal. The British Metropolis should surely send forth a respectable Quarterly Register, inferior to none; and the Editor pledges himself to use every effort to make it worthy of the distinguished patronage which it is now enjoying. The price will be found precisely at the same rate per sheet, as the monthly Medical Journals of London; and the Editor trusts that in no equal number of pages in any medical work in Europe, will a greater quantum of *concentrated matter* be seen, than in each number of this Journal. He hopes, therefore, that the work will be considered, what in fact it is, a very moderately priced publication.

*Albany, Piccadilly, Dec.* 27, 1818.

---

### MEDICAL PUPILAGE.

The Editor of this Journal has fitted up his Residence in the Albany as a place of study for himself and three private pupils, to the latter of whom practical information on professional subjects will be communicated, orally and by private Lectures. The Albany is in the centre of the Western School of Medicine, being equidistant from St. George's and the Middlesex Hospitals, and surrounded by the Medical, Surgical, and Anatomical Lectures. There is at present a vacancy for one Pupil. The Terms are *Thirty Guineas per Quarter, including Board.*

---

### ERRATUM.

In Dr. Porter's paper on Hydrocephalus, page 284, last line, omit *inverse*.

---

*Our next Number will be published on the First of April.*

*Additional Subscribers since last Quarter.*

Ayres, Mr. Mary-le-bone Street
Balantine, Mr. Middlesex Hospital
Best, Mr. Tavistock Street, Covent Garden
Blacklock, Mr. Surgeon, Dumfries
Boylston Medical Society, United States
Bruce, N. Esq. Surgeon, Royal Military College, Sandhurst
Birckhead, Mr. Lennox, Baltimore, United States
Burchell, Mr. Mare Street, Hackney
Bushell, Mr. Great Russell Street, Covent Garden
Calley, Mr. Surgeon, East Essex Militia, Colchester
Carmichael, Richard, Esq. Dublin
Clarke, Sir Arthur, Dublin
Clarke, Mr. Windmill Street, Haymarket
Daniels, Mr. Princes Street
Drew, Mr. James, Gower Street
Grabham, Mr. J. Surgeon, Rochefort
Hastings Dr. Charles, Physician to the Worcester Infirmary
Higgins, Mr. F. Little Russell Street, Covent Garden
Johnson, D. Esq. Torrington, Devon
Keenbyside, Mr. R. H.
Locock, Mr. Sackville Street
Lodge, Mr. Haymarket
M'Leod, Mr. John, R. N.
Merriman, Dr. Half Moon Street, Piccadilly
Mould, John, Esq. Surgeon, Oundle, Northamptonshire
Newenham, Mr. W. Farnham
Ogle, Richard, Esq. Great Russell Street, Bloomsbury
Paxton, Sir William, Piccadilly
Pool, John, Esq. Walton-upon-Thames
Powell, Mr. Newman Street
Rudge, Mr. Middlesex Hospital
Runciman, John, R. N.
Rutener, William, Esq. Bridgenorth
Smalley, Mr. Robert, Richmond Buildings
Sprague, J. H. Esq. Surgeon, North Curry, Somerset
Stedman, James, Esq. Guilford
Stokoe, Mr. Surgeon, St. Helena
Tedlie, Mr. Beresford, 85th Reg.
Valentine, Mr. John, London
Wake, Dr. Bartholomew, Somerset
Wallis, Mr. Charles, Lincoln's-Inn
White, Mr. B. at Mr. Brooke's
Whitney, Mr. 85th Reg.
Whitter, Dr. Fellow of the Royal College of Physicians, Bridge Street, Blackfriars
Wilmore, Mr. John, Surgeon, Worcester
Worcester Medical and Surgical Society, 2 Copies
Wright, Mr. George Street, Portman Square

---

☞ As a complete List of Subscribers will be given in our next Number, with the Index, Title Page, &c. all those friends of the Journal who wish their Names to be registered, are requested to send them to the Printer, *free of Expence.*

